# Global Changes in Local Protein Dynamics Reduce the Entropic Cost of Carbohydrate Binding in the Arabinose-binding Protein

**DOI:** 10.1016/j.jmb.2007.02.055

**Published:** 2007-05-04

**Authors:** Christopher A. MacRaild, Antonio Hernández Daranas, Agnieszka Bronowska, Steve W. Homans

**Affiliations:** Astbury Centre for Structural Molecular Biology and Institute of Molecular and Cellular Biology, University of Leeds, Leeds, LS2 9JT, UK

**Keywords:** ABP, arabinose-binding protein, HSQC, heteronuclear single quantum coherence, RDC, residual dipolar coupling, ligand binding, thermodynamics, NMR relaxation, molecular dynamics, periplasmic binding protein

## Abstract

Protein dynamics make important but poorly understood contributions to molecular recognition phenomena. To address this, we measure changes in fast protein dynamics that accompany the interaction of the arabinose-binding protein (ABP) with its ligand, d-galactose, using NMR relaxation and molecular dynamics simulation. These two approaches present an entirely consistent view of the dynamic changes that occur in the protein backbone upon ligand binding. Increases in the amplitude of motions are observed throughout the protein, with the exception of a few residues in the binding site, which show restriction of dynamics. These counter-intuitive results imply that a localised binding event causes a global increase in the extent of protein dynamics on the pico- to nanosecond timescale. This global dynamic change constitutes a substantial favourable entropic contribution to the free energy of ligand binding. These results suggest that the structure and dynamics of ABP may be adapted to exploit dynamic changes to reduce the entropic costs of binding.

## Introduction

An important goal of structural biology is to predict and manipulate the interactions between proteins and small-molecule ligands. To this end, extensive research efforts have been aimed at relating the known structure of protein–ligand complexes to the thermodynamics of that interaction. The success of these attempts has been limited, in part because of their neglect of the role of protein and ligand conformational dynamics in determining ligand-binding thermodynamics.

A common view of protein–ligand interactions sees them arising from the hydrophobic effect (the entropically favourable exclusion of water from hydrophobic surfaces) together with shape complementarity between the protein and ligand. In this view, protein–ligand interactions are expected to be driven by favourable changes in entropy. Recent results suggest closer scrutiny of this view is warranted, even for predominantly non-polar interactions.^1,2^ One can contrast this former view of protein–ligand interactions with one that considers the restriction in conformational degrees of freedom, which must necessarily be entailed by binding. From this perspective, an unfavourable change in entropy is expected to characterise these interactions. This requires a larger, favourable enthalpic contribution, which may arise directly from the protein–ligand interaction or from solvation effects.

The thermodynamic signature of protein–ligand interactions in this second view is typical of protein–carbohydrate interactions: entropic contributions to the interaction are typically large and unfavourable, and favourable enthalpic contributions drive the interaction. An example of such a system is the arabinose-binding protein (ABP). ABP is a member of the bacterial periplasmic binding protein family and serves as the initial component of the active transport system for the monosaccharides l-arabinose, d-fucose and d-galactose. Several members of this family, including ABP, have served as models for structural analysis of ligand-binding interactions,^3,4^ and have recently emerged as templates for design of novel ligand-binding proteins.^5^ ABP binds its ligands in the interface of two protein domains. The binding site displays an extensive network of H-bond and CH–π interactions characteristic of protein–carbohydrate association. Both structural and thermodynamic aspects of this interaction have been studied extensively,^4,6–9^ yet there is no clear understanding of the basis of either the large favourable binding enthalpy or the unfavourable entropy change on binding.

## Results and Discussion

### Entropic cost of binding

It is evident that formation of a stable complex between a protein and a ligand will involve the loss of entropy associated with the free diffusion of one component with respect to the other. The magnitude of these unfavourable contributions to the protein–ligand interaction may be only approximated; here, we take an estimate of the loss of ligand translational and rotational entropy from the work by Turnbull *et al* and Lundquist and Toone, which converge at approximately 25 kJ/mol for the equivalent free energy penalty.^10,11^ We note that this estimate is consistent with a number of others in the literature, for systems ranging from small polar molecules to folded and unfolded proteins.^12–15^

It is generally assumed that the bound ligand will adopt a single conformation, optimised to the structure of the binding site, and thus will experience a loss of entropy reflecting the loss of conformational flexibility of the ligand in solution. Likewise, the protein-binding site might be expected to undergo a loss of entropy as it adopts only that subset of available conformations that are conducive to binding. This loss of internal conformational entropy of the ligand is challenging to assess, particularly for a flexible ligand such as galactose, which displays complex conformational behaviour in solution. Nonetheless, on the assumption that ligand degrees of freedom are substantially “frozen” on binding, the entropic penalty arising from loss in degrees of freedom of the galactose hydroxyl rotors alone is likely to be ∼ 30 kJ/mol.^11^

**Figure 1:**
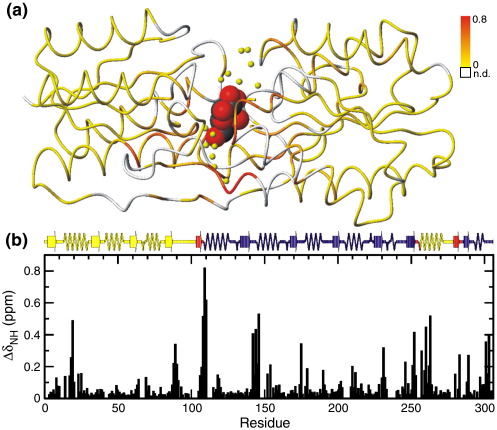
Backbone amide chemical shift changes for ABP on binding D-galactose. (a) Normalised amide chemical shift change (Δδ_NH_ = [Δδ_H_^2^+[Δδ_N_/5]^2^]^1/2^) is colour-mapped onto the backbone structure of the ABP–galactose complex (PDB Id 5ABP), with the colour scale shown. White represents residues that lack assignments in either apoABP or the complex. The ligand α-d-galactose is shown in the binding site as a space-filling model, and structurally conserved water molecules in the binding site are represented by yellow spheres. (b) The same data as in (a) plotted against the ABP sequence. Secondary structure elements are identified, with the N domain yellow, the C domain dark blue, and the domain hinge regions red.

The ABP binding site contains a significant number of tightly bound water molecules, which play a role in governing substrate specificity,^9^ and in maintaining the structure of the binding site. Examination of the structures of ABP in complex with arabinose (*1ABE*), fucose (*1ABF*) and galactose (*5APB*) reveals some 15 crystallographically resolved and structurally conserved water molecules within the binding site (within 10 Å of a ligand heavy atom and within 5 Å of heavy atoms of both protein domains; Figure 1). The entropic cost of confinement of water molecules has been estimated to be as much as 8 kJ/mol per water molecule.^16^ Even if the cost of confinement of the water molecules in the binding site of ABP is much less than this maximal value, it is clear that the overall cost of constraining these water molecules in the binding site will be substantial.

The experimentally observed entropy of the ABP–galactose interaction amounts to a *T*Δ*S*° of –61 kJ/mol at 308 K.^6^ Together, the entropic costs above exceed significantly this experimental entropy change on binding. Similar discrepancies, in which measured entropies of association are more positive (i.e. more favourable) than might be expected from first principles, have been known for at least 50 years.^17–19^ Most often, the discrepancy has been attributed to solvation effects, such as the hydrophobic effect or desolvation of charged groups.^17,18^ It has been noted, however, that these discrepancies might be explained by the introduction of new degrees of freedom in the complex, which did not exist in the interacting partners before interaction.^19^ We investigate this latter possibility by examining the contribution of protein dynamics to the entropy of interaction of ABP with its ligands.

### Assignment of apoABP

We have reported the determination of near-complete backbone resonance assignments for ABP in complex with its ligand, d-galactose.^20^ Assignments for apoABP were determined from these results, using an approach that combined conventional triple-resonance assignment strategies and ^1^H-^15^N heteronuclear single quantum coherence (HSQC) titrations of ABP with the fast-exchanging ligand 1-deoxy-galactose. Approximately 80% of the expected backbone resonances of apoABP were assigned.

From these assignments, a comparison was made of the chemical shifts of the backbone amide resonances of ABP in the apo state and in the complex (Figure 1). As expected, large chemical shift changes are seen in the binding site. In addition, large chemical shift changes are seen in the region linking the two domains of ABP, suggesting that ligand binding might be associated with a change in relative domain orientation in ABP. Domain reorientations on binding are observed in other members of the periplasmic-binding protein family, and have been proposed for ABP on the basis of the results of small-angle X-ray scattering and computational studies.^21,22^

Small chemical shift differences are seen at sites distal to the binding site and hinge region. These differences suggest that subtle changes in conformation or dynamics throughout the molecule occur on binding.

### Domain orientation of apoABP

Analysis of NMR relaxation for anisotropic molecules depends on knowledge of the molecular structure. It is therefore necessary to address the possibility of ligand-induced domain reorientation in ABP. We have done this using residual dipolar couplings (RDCs), which are sensitive to the average molecular orientation with respect to molecular alignment induced by a liquid crystalline solution.^23^ ^1^H-^15^N RDCs were measured for 127 backbone amides in regions of defined secondary structure of apoABP. The measured RDCs were initially compared to those predicted on the basis of the crystal structure of ABP in complex with galactose. Agreement was poor (RMSD = 13 Hz, *R*-factor = 65%), confirming that conformational change does indeed occur on ligand binding.

The extent of domain reorientation in ABP was determined using a structure calculation protocol in which the structure of each domain was minimised individually against the measured RDCs, followed by a simulated annealing procedure in which each domain was held rigid in its minimised conformation. This protocol was repeated 100 times with data generated by a Monte Carlo re-sampling of the experimental RDC data to assess the robustness of the protocol and the precision of the resulting structures. The agreement between the RDCs and the calculated structure is excellent, with an RMSD of 1.2 Hz and an *R*-factor of 6.0%. The quality of the structure is good, with 97% of residues in the core and allowed regions of the Ramachandran plot, and all residues showing correct peptide bond geometry. The backbone RMSD over the 100 structures derived from Monte Carlo re-sampling of the RDC data is 0.67 Å.

**Figure 2:**
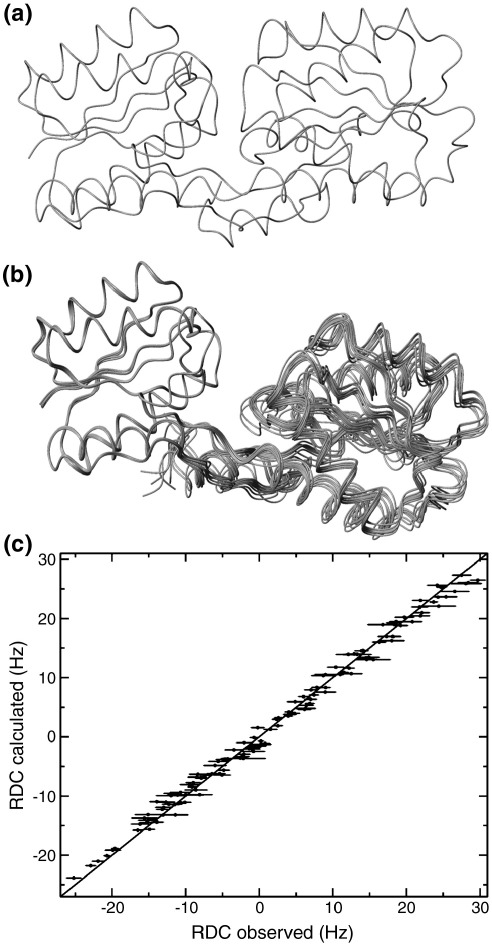
Residual dipolar coupling analysis of the domain orientation of apoABP. (a) Backbone trace of ligand-bound ABP from the X-ray crystal structure of the complex of ABP with galactose (PDB ID 5ABP). (b) Ensemble of ten backbone structures selected randomly from 100 structures calculated from data generated by a Monte Carlo re-sampling of the experimental RDC data. (c) The experimental RDC data plotted against the values back-calculated from the calculated structure closest to the average shown in (b).

The resulting ensemble of structures (Figure 2) showed apoABP to be “opened” by a domain hinge motion of ∼ 20° with respect to the crystal structure of the ABP–galactose complex. This represents a conformational change similar to those seen in other members of the periplasmic-binding protein family. In addition, small changes to individual domain structures are required for optimal agreement with RDCs. This supports the inference from chemical shift changes, that ligand binding has subtle conformational consequences throughout the protein.

### Dynamics of ABP

The contribution of protein dynamics to the ABP–galactose interaction was assessed by means of Lipari–Szabo analysis of nuclear magnetic relaxation.^24,25^ This approach yields information on the extent and timescale of molecular motions occurring faster than the rotational diffusion of the protein. The Lipari–Szabo formalism is relatively free of assumptions regarding the physical model describing the motion under investigation, requiring only that the dynamics be described by a Markov process and that the internal dynamics is uncorrelated with the global tumbling of the macromolecule. The results of the Lipari–Szabo analysis, in the form of an order parameter, can be interpreted in terms of the conformational entropy associated with the measured motions by means of a specific motional model. It has been shown that for a wide range of models, the functional dependence of entropy on the order parameter is similar, suggesting that changes in order parameter can be related to entropy change in a model-independent fashion.^26^

**Figure 3:**
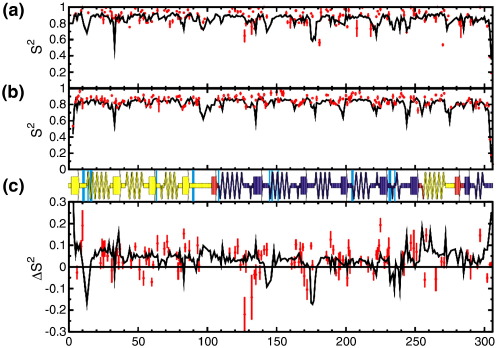
Backbone amide Lipari–Szabo order parameters for apo ABP and ABP bound to galactose. Generalised order parameters (*S*^2^) measured for (a) apoABP and (b) the ABP–galactose complex from NMR relaxation data (red) and calculated from MD simulation (black). (c) The change in order parameter (Δ*S*^2^ = *S*^2^_apoABP_–*S*^2^_ABPgal_) with secondary structure elements identified above. The N domain is yellow, the C domain dark blue, and the domain hinge regions red. Residues involved directly in the ligand interaction are highlighted in cyan.

We have measured relaxation data for 148 backbone amides of apoABP at three magnetic fields, and for 156 backbone amides of the ABP-galactose complex at two magnetic fields. The analysis of these data in terms of the Lipari–Szabo formalism yields order parameters that measure the extent of angular motion of individual amide bond vectors (Figure 3(a) and (b)). In both apoABP and the complex we see almost exclusively the large order parameters typical of the relatively rigid backbone of globular proteins. Remarkably, order parameters for apoABP are generally larger than for the ABP–galactose complex, indicating that pico- to nanosecond motions are more extensive in the complex than in the apo protein.

Owing to the size of ABP and the associated spectral complexity, many residues have been excluded from the analysis, because spectral overlap precludes accurate measurement of relaxation rates. For this reason, no experimental data are available for residues involved directly in binding. Thus, our observations reflect changes in dynamics remote from the binding site, yet caused by the protein–ligand interaction. Indeed, the observed changes are seen to be distributed throughout the protein, albeit with a slight bias towards the N domain (in the sense that larger dynamic changes are seen preferentially in this domain) (Figure 3(c)).

To confirm and further explore the basis of this result, we have performed molecular dynamics simulations for ABP in the apo state and in complex with galactose. By several measures, we see significant increases in backbone dynamics in the complex as compared with the apo protein. RMS deviations from the average structure for both the N and C domains are significantly larger for the ABP–galactose complex than for apoABP. In addition, fluctuations of backbone heavy-atom positions across the trajectory are generally larger in the complex than in the apo protein (data not shown). Furthermore, these dynamic changes are seen to be more pronounced in the N domain than the C domain, consistent with the experimental observations.

To make a direct comparison between the experimentally observed order parameters and the simulations, backbone amide order parameters have been calculated from the molecular dynamics trajectory.^27^ We see good agreement between measured and calculated order parameters, albeit with a small tendency for the simulation to underestimate the experimental values (Figure 3(a) and (b)). Underestimation of measured order parameters by molecular dynamics simulations has been a common observation in previous comparisons between molecular dynamics simulation and NMR relaxation measurements, particularly in loops and other flexible regions.^27–30^ This small systematic discrepancy is of little importance in this instance, as we are interested principally in the difference in order parameter between the two states. Indeed, the changes in order parameter upon ligand binding reproduce the measured changes excellently, showing an approximately uniform decrease in order parameter upon binding across much of the protein (Figure 3(c)). The average change in order parameter (*S*^2^_apoABP_–*S*^2^_ABPgal_) determined by the two methods is identical, at 0.038; the uncertainty on this average value, derived by propagation of experimental uncertainties in the individual order parameters, is 0.005. The correlation coefficient between the experimental and simulated order parameters is 0.83 for the complex protein and 0.47 for the apo-protein.

**Figure 4:**
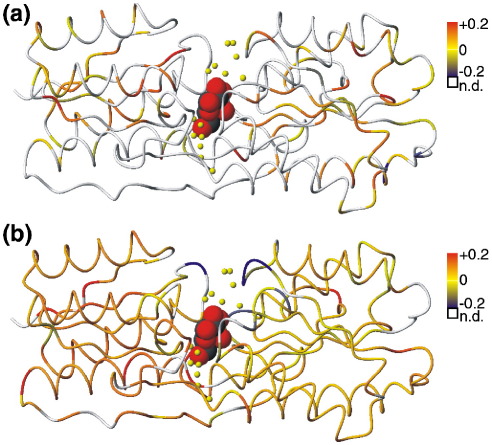
The change in backbone amide order parameter (Δ*S*^2^ = *S*^2^_apoABP_–*S*^2^_ABPgal_) from (a) NMR and (b) MD is colour-mapped onto the backbone structure of the ABP–galactose complex. The colour scale for Δ*S*^2^ values is shown. Residues for which Δ*S*^2^ could not be determined due to peak overlap in one of the NMR data sets or failure to converge in one of the MD simulations are white. α-d-Galactose is shown in the binding site as a space-filling model, and structurally conserved water molecules in the binding site are represented by yellow spheres.

As well as validating our experimental results, the molecular dynamics simulations reveal details of dynamic changes in regions that could not be measured experimentally. Notably, the simulations reveal complex changes in dynamics in the binding site (Figure 4(b)). Several residues in the binding site display increases in flexibility upon binding, consistent with the trend seen throughout the rest of the molecule. Other binding site loops display a decrease in flexibility, more in keeping with the intuitive expectation that ligand binding will reduce the conformational freedom of binding site residues.

A significant assumption entailed by the Lipari–Szabo analysis performed here is that the rotational diffusion of the protein can be fully characterised by a single diffusive process uncorrelated with the internal motions under investigation.^24^ This assumption is generally robust for globular, single-domain proteins, but might be called into question in the case of ABP, where flexibility of the domain hinge may result in a complex interaction between local protein conformation and rotational diffusion.

In the case of the ABP–galactose complex, we assume such flexibility to be insignificant, as the ligand binds in and stabilises the domain interface. No such assumption can be made for apo ABP, however. In an attempt to assess the influence of inter-domain flexibility in apoABP on our results, we have repeated the Lipari–Szabo analysis, assuming each domain undergoes independent rotational diffusion. The expectation is that the apparent rotational diffusion of each domain should differ from that of the molecule as a whole if significant inter-domain flexibility is present. The best-fit diffusion tensors arising from the analysis of the two individual domains are very similar, but are slightly different from the diffusion tensor derived from the whole protein (Table 1). This suggests that inter-domain flexibility has only a minor influence on the measured relaxation rates. The orientation of the diffusion tensors for each domain is consistent with the RDC-derived structure of apo ABP, highlighting a consistency between the RDC and relaxation data that would not be expected if the relaxation data were influenced substantially by domain motions. Importantly, the Lipari–Szabo dynamic parameters derived from this analysis are identical, within error, with those of the initial analysis.

Furthermore, we note that inter-domain flexibility can, to a first approximation, be accounted for by the so-called extended Lipari–Szabo treatment.^25^ Here, inter-domain flexibility is treated by the slow dynamic component (*S*^2^_s_, τ_s_), while internal motions are treated by the fast dynamic component (*S*^2^_f_).^31,32^ Because the reported overall order parameter is the product of the fast and slow order parameters (*S*^2^_LZ_ = *S*^2^_f_ S^2^_s_), the effect of inter-domain flexibility (*S*^2^_s_ < 1) will always be the overestimation of the extent of internal motions. As such, if domain flexibility in apoABP does influence our analysis, its effect will be the underestimation of the increase in internal dynamics upon ligand binding. In this context, it is of interest to note that there are more residues that are best treated by the extended Lipari–Szabo model in apoABP than there are in the ABP–galactose complex (Table 2).

Additional evidence that inter-domain motions have little impact on our overall results is obtained from the MD simulations. The contributions of rotational diffusion to the simulated dynamics are removed in the conventional way by alignment of the protein at each snapshot to a single reference structure. Alternatively, contributions of both rotational diffusion and inter-domain flexibility can be removed by separately aligning a single domain to a reference structure before analysis of the internal dynamics of that domain. There is no significant difference between these two approaches in terms of the calculated order parameters (data not shown). This reflects the small amplitude of the domain flexibility observable in the production phase of the apo ABP trajectory. On this basis, it is likely that domain flexibility is either very small or occurs on a timescale slower than is detectable in the 20 ns simulations analysed here. Given the anomalously low viscosity of the TIP3P water model used in these simulations, the rate of domain flexibility in the simulation is likely to significantly overestimate the *in vitro* rate. Thus, the failure to detect significant domain flexibility suggests that the relevant timescale is slower than the overall rotational diffusion of ABP (∼ 15 ns), and therefore is not expected to contribute to the measured relaxation data.

A further caveat of the analysis of NMR relaxation data concerns its insensitivity to dynamics occurring on a timescale similar to or slower than the rate of rotational diffusion. Given this insensitivity, it is conceivable that the apparent increase in flexibility of ABP on ligand binding may in fact represent a shift in the dynamic timescale. This would imply that the dominant timescale for motions in apoABP is slower than ∼ 15 ns, but that this shifts to a much faster timescale on ligand binding. There are two reasons to discount this interpretation of our data. Firstly, we consider it most likely that a timescale shift of the type considered here would result in a shift from predominantly simple Lipari–Szabo models in the apo-protein to a preponderance of complex models in the ligand-bound form, as new observable motional modes are introduced by the change in timescale. In fact, precisely the opposite trend is observed (Table 2). Secondly, we note that there is no evidence of extensive slow timescale motions in our molecular dynamics studies. The presence of dynamics on this timescale would be reflected by inadequate sampling over the course of our 20 ns trajectories. In fact, using the convergence test proposed by Best *et al.*,^29^ the calculated order parameter is judged to have converged in all but nine residues in apo ABP, and in all but four residues in the ABP–galactose complex. All residues for which dynamics are not converged are in flexible loops in ABP.

**Figure 5:**
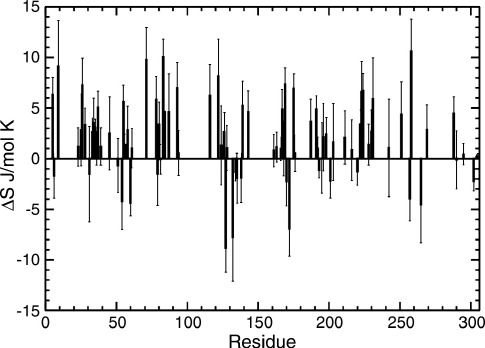
The per-residue change in conformational entropy (Δ*S*_conf_ = *S*_conf,apoABP_–*S*_conf,ABPgal_) calculated from experimental order parameters as described.^26^

To understand the thermodynamic implications of the observed changes in dynamics, we exploit the relationship between Lipari–Szabo order parameter (*S*^2^_LZ_) and conformational entropy (*S*_conf_) derived by Yang and Kay.^26^ Of the 306 residues of ABP, we determined an entropy change associated with the measured change in pico- to nanosecond dynamics for 84 residues. Entropy change for the remaining residues could not be determined either because *S*^2^_LZ_ could not be determined reliably for both the apo and ligand-bound states (203 residues), or because *S*^2^_LZ_ > 0.95 in one or both states (19 residues). These data are plotted in Figure 5. The average entropy change per residue is 2.0(± 0.3) J/mol K. This amounts to an overall entropy change of 170(± 30) J/ mol K for the measured residues (*T*Δ*S*_conf_ = 52(± 9) kJ/mol at 308 K). If it is assumed that this average entropy can be applied to all un-measured residues, and that dynamics changes for each residue are not correlated, the total entropy change from changes to pico- to nanosecond motion on ligand binding amounts to 610(± 120) J/mol K (*T*Δ*S*_conf_ = 188(± 37) kJ/mol at 308 K). Clearly, this latter value amounts to an overestimate, as the assumption of un-correlated motion is not likely to hold for all residues of the protein. It is evident, however, that the entropy change associated with the change in pico- to nanosecond dynamics contributes significantly to the favourable free energy of binding, and in effect amounts to protein dynamics paying some of the unfavourable entropic cost of ligand binding.

The experimental data available for this system is limited to probes of backbone dynamics. Given the good agreement between experiment and the molecular dynamics simulations, it is perhaps reasonable to infer something of the side-chain dynamics from these simulations. We observe considerably more variability in side-chain order parameters than is evident for the backbone, consistent with findings in other proteins. Furthermore, there is much greater variability in the change in order parameter observed upon ligand binding. Despite this, the changes in side-chain dynamics are broadly similar to those seen for the backbone, with the majority of residues showing a small increase in flexibility on ligand binding. As fast side-chain dynamics are correlated with local backbone dynamics only weakly, this suggests an additional source of favourable entropy change accompanying binding. A number of residues around the binding site show larger changes in dynamics on binding, reflective of both increases and decreases in flexibility. These changes reflect similar heterogeneous dynamic changes seen in the protein backbone of the loops that comprise the binding site.

One particularly surprising aspect of these results is the dispersed and approximately uniform nature of the change in dynamics. This is unexpected, because fast motions in proteins, such as the pico- to nanosecond dynamics under study here, are almost exclusively local in character, with few if any correlations over distances longer than a few ångström units.^30^ One plausible explanation for the uniform nature of the observed effect is that it is an artefact arising from a systematic error in the determined rotational diffusion tensor for the apo and/or holo protein. To exclude this possibility, we have repeated the analysis using the approach described by Schurr *et al*., which fits the relaxation data for each residue individually, with a so-called local τ_m_ term to account for rotational diffusion.^33^ In this analysis, the sets of dynamic parameters determined for each residue are statistically independent of one another, and are independent of any structural model for the protein. We find no significant difference between order parameters derived from this analysis and those obtained from the conventional analysis, where rotational diffusion is treated as a global parameter in the form of an anisotropic diffusion tensor. Furthermore, the local τ_m_ values determined in this analysis are fully consistent with the diffusion tensor determined conventionally.

We conclude that the results described here are robust with respect to the models of protein structure and rotational diffusion used in the analysis. These results therefore indicate a coordinated global change in local dynamics, initiated by the localised event of ligand binding. The physical basis for such a change is not clear, but it is not without precedent in the literature: several studies have identified favourable changes in pico- to nanosecond backbone dynamics on ligand binding to be similarly dispersed throughout the protein.^34–36^ Other studies have identified broadly dispersed changes in dynamics of contrasting sign, such that favourable changes in one part of a protein appear to compensate for unfavourable dynamic changes elsewhere in the protein.^37,38^

It has been appreciated for some time that protein dynamics might potentially mediate aspects of protein function including allosteric regulation^39^ and catalysis.^40^ Experimental evidence for such a functional role, principally from NMR spectroscopy, has been accumulating.^41,42^ These results raise the possibility that proteins may be adapted evolutionarily to exploit internal dynamic processes to functional ends. The results described here, together with previous observations,^34–38^ suggest that proteins may have evolved finely tuned networks of dispersed dynamic interactions that regulate the thermodynamics of protein–ligand interactions. We are actively exploring the mechanism of this phenomenon in ABP, and the contribution of other degrees of freedom to the binding entropy, particularly ligand and protein side-chain dynamics.

## Materials and Methods

### Protein expression and purification

ABP was expressed as essentially as described,^6,20^ except that the *Escherichia coli* host strain was BL21(DE3), and the growth medium was M-9 minimal medium, supplemented with BME vitamins (Sigma). For the production of ^15^N-labelled protein, ^15^NH_4_Cl was the sole nitrogen source, and for production of 50% ^2^H, ^13^C, ^15^N protein, the medium was 50% ^2^H_2_O and the carbon and nitrogen sources were [U-^13^C, 50% ^2^H]glucose and ^15^NH_4_Cl, respectively. Purification was as described.^6,20^

### NMR spectroscopy

All NMR samples contained approximately 1 mM ABP in 20 mM potassium phosphate (pH 7.0), 3 mM sodium azide, 0.1 mM EDTA, 10% ^2^H_2_O. For studies of the complex of ABP with galactose, the sample contained approximately 5 mM d-galactose. Except where noted, NMR experiments were performed at 308 K on Varian INOVA spectrometers operating at 500 MHz, 600 MHz or 750 MHz ^1^H frequency and equipped with room-temperature triple-resonance *z*-gradient probes. The transverse relaxation optimized spectroscopy (TROSY)-HNCA of apoABP was acquired at 600 MHz with 16 transients and 1024, 60 and 40 complex points and spectral windows of 7510, 2800 and 4000 Hz in the ^1^H, ^15^N, and ^13^C dimensions, respectively.

RDCs were measured at 303 K and 750 MHz using 3.5% (w/v) C5E12 alkyl-poly(ethylene glycol)/hexanol (1:0.96 molar ratio)^43^ as an alignment medium. ^1^H-^15^N one-bond couplings in the presence and in the absence of the alignment medium were measured using the ^1^*J*_NH_ modulated HSQC described by Tjandra *et al*.^44^ The dephasing delay was varied in 1 ms steps from 6 ms to 20 ms, with two points duplicated for error estimation. Each increment was acquired with 40 transients, 1024 × 128 complex points and spectral windows of 6500 × 3200 Hz. Residues for which fewer than two zero-crossings could be clearly identified were excluded from further analysis. Uncertainty in the measured couplings was estimated using multiple fits using a bootstrap resampling procedure.^45^

Backbone amide relaxation parameters (^15^N R_1_ and R_2_, and the ^1^H-^15^N heteronuclear NOE) were measured using the pulse sequences reported by Farrow *et al*.^46^ Typical relaxation delays were 10.7 ms, 53.3 ms, 107 ms, 213 ms, 426 ms, 640 ms, 906 ms, 1230 ms, 1600 ms and 2130 ms for *R*_1_ and 12.4 ms, 16.6 ms, 24.8 ms, 33.1 ms, 49.7 ms, 66.2 ms, 82.8 ms, 99.4 ms and 133 ms for *R*_2_. Duplicate measurements were made for at least two points in each series for estimation of experimental error. In the *R*_2_ experiment, dummy Carr–Purcell–Meiboom–Gill sequences were applied before the recovery delay to compensate for sample heating caused by radio-frequency pulses, and all experimental series were acquired fully interleaved. Typically, 32 transients were acquired for *R*_1_ and *R*_2_ measurements, and 64 for the NOE, giving a total experimental time of approximately 60 h for the three experiments at each field. Measurements were made at 500 MHz, 600 MHz and 750 MHz for apo ABP, and at 500 MHz and 600 MHz for the ABP–galactose complex.

### Spectral assignment

Resonance assignments for the ABP–galactose complex have been determined.^20^ Many peaks in the ^1^H-^15^N HSQC show small chemical shift changes between the apo and complexed states, permitting partial apoABP assignments to be determined on the basis of those for the complex. For assignment of ^1^H-^15^N HSQC resonances that do move on binding ligand, a titration of ABP with 1-deoxygalactose was used. 1-deoxygalactose binds ABP with an affinity four orders of magnitude less than that of galactose, and is in the fast-exchange regime. Assignments for a number of resonances remained ambiguous at this stage, owing to substantial crowding of the ^1^H-^15^N HSQC spectrum. These remaining ambiguities were largely resolved by means of *i* to *i*–1 connectivities derived from an HNCA spectrum of apoABP.

### Domain orientation of apoABP

ABP is composed of two structural domains, the N domain (residues 1–103 and 257–277), and the C domain (residues 110–253 and 286–306). The remaining residues form a three-stranded linker between the domains, which retains some flexibility in the absence of ligand.^21,22^ The relative orientation of the two domains of apoABP was determined using ^1^H-^15^N RDCs. An initial estimate of the tensor describing molecular alignment of apoABP in 3.5% (v/v) C5E12/hexanol was obtained from the histogram of experimental RDCs.^47^ A starting structure was derived from the structure of ABP in complex with galactose (PDB ID *5ABP*) by Powell minimisation of each domain individually against the measured RDCs.^9^ From this structure, domain orientation is determined by rigid-body/torsion angle simulated annealing in which both domains are held rigid. All calculations were performed using the IVM module of Xplor-NIH.^48^ The annealing protocol comprised an initial 3 ps/1000 steps of dynamics at 2000 K, after which the temperature was reduced to 25 K in 12.5 K steps, with 0.3 ps/100 steps of dynamics performed at each temperature. Default Xplor-NIH parameters were used throughout, and force constants were ramped using default values. Square-well potentials were used for all RDC constraints.

### Lipari–Szabo analysis of relaxation data

Backbone amide relaxation parameters were analysed in terms of the extended Lipari–Szabo formalism,^24,25^ using the software relax.^49,50^ The ^15^N CSA was set at −170 ppm and the effective N–H bond length was 1.02 Å. Initial estimates for the rotational diffusion tensor of both apoABP and the ABP–galactose complex were obtained from the ratio of longitudinal and transverse relaxation rates.^51^ Residues that experience complex internal motion or Rex contribution were identified as described and excluded from this diffusion tensor estimation.^52^ For ABP–galactose, an axially symmetric diffusion tensor produces an optimal fit, while the fit to the apo ABP data is improved significantly by the use of a fully anisotropic diffusion tensor. Similar results were obtained using data collected at each field strength.

Using these estimates, parameters of the extended model-free formalism were optimised for each residue individually, and the best parameter set identified by AIC model selection.^49^ All parameters, including the diffusion tensor, were then optimised. This process was repeated until the solution converged. The final optimised diffusion tensors are presented in Table 1, and the final dynamic parameter sets in Table 2.

Additional analyses were performed as described.^33^ Here, the contribution to relaxation due to overall rotational diffusion is treated as a mono-exponential component to the correlation function, with a time constant determined individually for each residue; the so-called local τ_m_. In this analysis, the dynamic parameters and local τ_m_ for each residue are optimised individually, and the optimal parameter set selected by AIC.^49^ We did not include chemical exchange (*R*_ex_) contributions to *R*_2_ in this analysis, owing to the tendency for un-physically low local τ_m_ values to be compensated by artefactual *R*_ex_ contributions and low order parameters, particularly in the case of the ABP–galactose complex, where only two *R*_2_ values are available for each residue.

Changes in conformational entropy associated with the observed changes in Lipari–Szabo order parameters were determined using the relationship described by Yang and Kay.^26^ This relationship is essentially independent of motional model for *S*^2^_LZ_ ≤ 0.95, so only residues consistent with this criterion in both apoABP and the complex have been included in considerations of entropy change. Uncertainty in the calculated entropy change deriving from experimental uncertainty in order parameters was determined by standard error propagation methods.

### Molecular dynamics

The simulations were carried out using AMBER 8,^53^ with the Cornell *et al*. force field.^54^ Initial coordinates of both holo and apo forms were based on the crystal structure of the ABP–galactose complex (PDB code *5ABP*).^9^ The structures were processed by the XleaP module of AMBER, and the hydrogen atoms were added to the system. The structure of β-d-galactopyranose was optimised *ab initio* using the Gaussian 98 program† with the HF/6-31G* basis set, and RESP charges were generated and fitted.^55^ The ligand molecule was parameterised using GLYCAM force field.^56^ The models were subjected to short (1000 cycles) energy minimisation.

Protein models were then immersed in periodic TIP3P water boxes. Approximately 5500 water molecules were added to each system. Simulations were carried out under NPT conditions at 300 K, using the particle mesh Ewald technique with 12 Å non-bonded cutoff and 2 fs time-step.^57^ SHAKE constraints with a tolerance of 10^− 8^ Å were applied to all hydrogen atoms during MD simulations to eliminate the fastest X-H vibrations and allow a longer simulation time-step. Translational and rotational centre-of-mass motion was removed every 10 ps. Equilibration started by 20 000 cycles of energy minimisation, with the atomic positions of the solute molecule restrained. It was followed by 100 ps of MD simulations, where the system was heated gently to 300 K and the constraints were released gradually (from 100 kcal/(mol·Å^2^) applied initially). The further equilibration took 4.9 ns.

The production period took 20 ns for both systems. The coordinates were saved every 2 ps of MD simulation.

Generalised order parameters were calculated from the trajectory of individual back-bone amide bond vectors as:^27^

(1) $$S_{LZ}^{2}=3/2[<x^{2}{>}^{2}+<y^{2}{>}^{2}+<z^{2}{>}^{2}+2<xy{>}^{2}+2<xz{>}^{2}+2<yz{>}^{2}]-1/2$$

where *x*, *y* and *z* are components of a unit vector along the amide bond, and angular brackets denote the time-average over the trajectory. Convergence of the dynamics of interest is tested using the approach described by Best *et al.*:^29^ a cumulative time function *S*^2^_LZ_(τ) is defined using equation (1), with the time averages taken from *t* = 0 to *t* = τ. This function was evaluated for 100 equally spaced time-points across the trajectory. The trajectory was deemed to have converged if the difference between the maximum and minimum values of this function over the final 50 time-points (i.e. the final 10 ns of the trajectory) was less than 0.05. Any residue judged not to have converged was excluded from the analysis.

## Figures and Tables

**Table 1:** Rotational diffusion tensors from the optimisation of Lipari–Szabo formalism

	Tensor symmetry	*D*_x_ (s^− 1^)^a^	*D*_y_ (s^− 1^)^a^	*D*_z_ (s^− 1^)^a^	τ_m_ (s)^b^	*D*_a_ (s^− 1^)^c^	*D*_r_^d^
ApoABP	Anisotropic	9.68(± 0.10) × 10^6^	1.03(± 0.01) × 10^7^	1.31(± 0.01) × 10^7^	1.51(± 0.002) × 10^− 8^	3.15(± 0.08) × 10^6^	0.0973 ± 0.015
ApoABP–N domain	Anisotropic	8.83(± 0.02) × 10^6^	1.02(± 0.02) × 10^7^	1.47(± 0.01) × 10^7^	1.48(± 0.003) × 10^− 8^	5.21(± 0.13) × 10^6^	0.134 ± 0.017
ApoABP–C domain	Anisotropic	8.21(± 0.02) × 10^6^	9.59(± 0.02) × 10^6^	1.49(± 0.01) × 10^7^	1.53(± 0.003) × 10^− 8^	5.98(± 0.12) × 10^6^	0.116 ± 0.012
ABP-gal	Axially symmetric				1.35(± 0.003) × 10^− 8^	4.50(± 0.12) × 10^6^	–

a *D*_x_, *D*_y_ and *D*_z_ are the diffusion constants about the principal axes of the anisotropic tensor. Uncertainties are estimated using Monte Carlo resampling of the experimental data, with the dynamic parameters treated as fixed at their optimal values.

b Average rotational correlation time. τ_m_ = (2(*D*_x_+*D*_y_+*D*_z_))^− 1^ for anisotropic tensor, and τ_m_ = (2(*D*_∥_+2*D*_⊥_))^− 1^ for axially symmetric tensor. *D*_∥_ is the diffusion constant about the unique axis of the axially symmetric tensor, and D_⊥_ is the diffusion constant perpendicular to the unique axis.

c Anisotropy *D*_a_ = *D*_z_–(*D*_x_+*D*_y_)/2 for anisotropic tensor, and *D*_a_ = *D*_∥_–*D*_⊥_ for axially symmetric tensor.

d Rhombicity *D*_r_ = (*D*_y_–*D*_x_)/2*D*_a_ for anisotropic tensor, and not defined for axially symmetric tensor.

**Table 2:** Distribution of fits of amide-bond vectors to various Lipari–Szabo motional models

	Model 1^a^	Model 2^b^	Model 3^c^	Model 4^d^	Model 5^e^
apoABP	54	17	12	5	60
ABP-gal	62	28	13	11	41

a Simple internal motion faster than ∼ 10 ps. Fitted parameters are {*S*^2^_LZ_}.

b Simple pico- nanosecond internal motion. Fitted parameters are {*S*^2^_LZ_, τ}.

c As model 1 with chemical exchange contribution to *R*_2_. Fitted parameters are {*S*^2^_LZ_, *R*_ex_}.

d As model 2 with chemical exchange contribution to *R*_2_. Fitted parameters are {*S*^2^_LZ_, τ, *R*_ex_}.

e Extended Lipari–Szabo formalism:^25^ both fast (< 10 ps) and slow (about nanosecond) motions are present. Fitted parameters are {*S*^2^_LZ_, *S*^2^f, τ_s_}.

## Footnotes

† Frisch, M. J. *et al*. (1998). Gaussian 98 (Revision A.9). Gaussian, Inc., Pittsburgh, PA.
